# Therapeutic Intervention for Osteoporosis of Surgical Cases With Non-Vertebral Fractures for Three Years in a Newly Established Rural Hospital in Japan

**DOI:** 10.7759/cureus.63864

**Published:** 2024-07-04

**Authors:** Masanori Nakayama, Yasuhiro Kiyota, Naoki Watanabe, Kento Yamanouchi, Satoshi Nakamura, Kenichiro Takeshima, Haruki Funao, Shigeto Ebata, Mitsuru Yagi

**Affiliations:** 1 Orthopedic Surgery, International University of Health and Welfare, Narita, JPN

**Keywords:** proximal humerus fracture, distal radius fracture, proximal femoral fracture, aging society, non-vertebral fracture, newly established hospital, osteoporosis

## Abstract

Objectives

Our hospital was newly opened in the spring of 2020 in a rural area of Japan, with a remarkably developing aging society and population decline. This study aimed to clarify and evaluate the practice of osteoporosis care in our hospital for three years since its establishment. We report a retrospective review of therapeutic interventions for osteoporosis for patients who underwent surgical treatment for non-vertebral fragility fractures in our hospital.

Methods

We evaluated the practice of osteoporosis intervention in patients who underwent surgery for proximal humerus fractures (PHFs), distal radius fractures (DRFs), or proximal femoral fractures (PFFs) from April 2020 to the end of March 2023.

Results

There were 115 surgical cases with non-vertebral fractures (10 patients with PHF, 41 patients with DRF, and 64 with PFF). Among the patients who had received osteoporosis treatment at other hospitals before the injury, only 15 (13.0%) patients had been administered therapeutic intervention for osteoporosis by other clinics or hospitals. Also, 82 (71.3%) patients were newly diagnosed with osteoporosis in our hospital after surgery according to the Japanese osteoporosis guideline. New postoperative osteoporosis interventions were administered to 39 (47.0%) patients, of which the rate was higher than the previous reports in Japan. While there was no significant difference between upper limb fracture and PFF in the percentage per young adult mean of spine areal bone marrow density (aBMD), the femoral neck aBMDs in the upper limb fracture group were significantly higher than in the PFF group. The serum total P1NP levels were significantly lower and the 25(OH)D levels were also greater in the upper limb fracture group than in the PFF group, whereas the serum TRACP-5b levels were not significantly different between the two groups. Two (1.7%) patients were affected with secondary fractures during the study period.

Conclusions

The rates of therapeutic intervention for osteoporosis of patients with non-vertebral fractures, especially in those with upper limb fractures, in our hospital were considered to be greater than those in the previous reports. However, the intervention rate for patients with PFFs was not much, and there was still room for improvement in our hospital concerning osteoporosis diagnosis and treatment.

## Introduction

Recently, the aging of the population has become a major issue worldwide [1], especially in Japan [2,3], where the cost of medical care for diseases and trauma has skyrocketed and become a very important social problem [1]. In particular, the incidence of fragility fractures caused by osteoporosis, which is common among elderly people, has increased [4], and diagnostic and therapeutic interventions for osteoporosis aimed at fracture prevention are urgent issues in Japanese healthcare [5,6]. Moreover, since the incidence rate of the second or more fractures after fractures was much higher than the incidence of the first fracture [7,8], therapeutic interventions for osteoporosis to prevent the first fracture are very important.

According to the recent large Japanese studies, however, the percentage of patients who underwent bone marrow density (BMD) tests after surgery for distal radius fracture (DRF), one of the most common fragility fractures, was very low (126/1445, 8.7%), and the rate of therapeutic intervention for osteoporosis was also low (193/1445, 13.4%) [2]. The rate of osteoporosis intervention after proximal femoral fracture (PFF) surgery, which is also one of the most common fragility fractures, is similarly low, at 436/2,328 (18.7%) [9]. Such a low rate of osteoporosis treatment was found to be more remarkable in rural areas than in urban areas in Japan [10,11], reflecting the reality of the underserved medical care in rural areas in Japan.

In Japan, new medical schools had not been established for around 40 years by the government to avoid medical doctors’ excessing compared with the population [12]. Two medical schools were established in 2016 and 2017, and our medical school is the newest one. Our hospital opened in the spring of 2020 as the main hospital of our medical school. Since our hospital is entirely new and not inherited from any existing hospital, recruiting patients has been difficult for several months right after opening. Our orthopedic department developed a new effective system for diagnosing and treating osteoporosis to take advantage of the newly opened hospital. First, a special outpatient for osteoporosis treatment has been established since the beginning of our hospital. Second, testing equipment for osteoporosis, including a dual-energy X-ray absorptiometry (DEXA) bone densitometer (PRODIGY Fuga®, GE HealthCare, Chicago, USA), was installed immediately after its establishment. Third, educational workshops and lectures on osteoporosis diagnosis and treatment for young physicians and medical staff were held regularly, and seminars on osteoporosis were regularly held by our hospital and the nearby clinics and hospitals cooperatively. Lastly, the BMD test and blood test of bone turnover biomarkers have been fixed in our clinical paths at the beginning.

The present study aimed to clarify the practice of osteoporosis care at a newly established hospital in a rural area of Japan, which has a remarkably developing aging society and population decline. We report a retrospective review of therapeutic interventions for osteoporosis performed at our hospital for patients who underwent surgical treatment for non-vertebral fragility fractures, including proximal humerus fracture (PHF), DRF, and PFF, for three years since our hospital opening.

## Materials and methods

Patients

We corrected patients who underwent surgery for PHF, DRF, or PFF at our hospital from April 2020 to March 2023, three years since the opening. Patients with fractures due to high-energy trauma, those with pathological fractures such as those resulting in the metastasis of malignant tumors, or child/adolescent patients with fractures were excluded from this study. This study was approved by the Ethical Committee of our institute (20-Nr-014).

Evaluated outcomes

We investigated the following clinical parameters: 1) the presence of prior osteoporosis treatment before the fracture, 2) whether BMD tests (measuring at the lumbar spine and femoral neck) and blood tests with bone turnover biomarkers measurements were performed at our hospital after the fracture, and 3) whether an osteoporosis diagnosis was made and whether the new treatment was started postoperatively.

The additional evaluated items were the score of the percentage of BMD per young adult mean (YAM) and the values of major bone turnover biomarkers measured at the hospital, such as total procollagen type I propeptide (P1NP), tartrate-resistant acid phosphatase 5b (TRACP-5b), and 25-hydroxy-vitamin D (25(OH)D), limited to patients who underwent the examinations. In addition, we investigated details of the medications for osteoporosis and whether a secondary fracture occurred during the follow-up period.

Statistical analysis

Clinical characteristics were compared using Fisher's exact test for categorical variables and Student’s t-test for continuous variables. A p-value of <0.05 was considered to indicate statistical significance. All the statistical analyses were performed using EZR (Saitama Medical Center, Jichi Medical University, Japan), which is a graphical user interface for R (The R Foundation for Statistical Computing, Vienna, Austria).

## Results

The characteristics of the included patients are shown in Table 1. During the observation period, 115 (84 women and 31 men; mean age of 75.1 years) patients underwent surgery for non-vertebral fragility fractures, including 10 patients with PHF, 41 patients with DRF, and 64 patients with PFF, and the mean postoperative observation period was 11.8 months. Patients with upper limb fractures were younger than those with PFFs, and only 15 (13.0%) patients were treated for osteoporosis at other clinics or hospitals before the fractures. Additionally, 50 (78.1%) patients with PFFs were transferred to rehabilitation hospitals after surgeries and 13 (26.0% per patients transferring and 20.3% per all patients with PFFs) were back to our outpatient clinic, and none of the patients with upper limb fractures were transferred to other hospitals after surgeries.

**Table 1:** Characteristics of the participants in this study The data are presented as the means (minimum-maximum), numbers, or percentages. *Between upper limb fracture and lower limb fracture. PHF, proximal humerus fracture; DRF, distal radius fracture; PFF, proximal femoral fracture

	Total	Upper limb fracture			PFF	p-Value*	t-Value
		Total	PHF	DRF			
Number of patients	115	51	10	41	64	-	-
Women, number (%)	84 (73.0)	37 (72.5)	7 (70.0)	30 (73.2)	47 (73.4)	0.999	-
Age (mean), years	75.1 (43-97)	68.7 (43-94)	72.4 (60-88)	67.8 (43-94)	80.2 (54-97)	<0.001	6.27
Postoperative observation period (mean), months	11.8 (0.5-46)	14.2 (1-35)	10.5 (1-35)	15.1 (1-35)	9.9 (0.5 -46)	0.0291	-2.21
Previous osteoporosis treatment done, number (%)	15 (13.0)	4 (7.8)	2 (20.0)	2 (4.9)	11 (17.2)	0.171	-

The clinical outcomes are shown in Table 2. BMD tests were performed for 67 (58.2%) patients and blood tests with bone turnover biomarkers were performed for 47 (40.9%) patients after the fracture, and 82 (71.3%) patients were newly diagnosed with osteoporosis after surgery according to the Japanese osteoporosis guideline [13]. New osteoporosis interventions were postoperatively administered to 39 (47.0%) patients, including 15 (83.3) patients with upper limb fractures (two, 100%, patients with PHFs and 13, 81.3%, patients with DRFs) and 24 (37.5%) patients with PFFs. The rate of new administration of osteoporosis treatment in patients with upper limb fractures was significantly higher than in patients with PFFs (p<0.05). Among the patients who underwent the BMD test, the mean percentage of spine areal BMD (aBMD) per YAM (%YAM) was 86% and that of the femoral neck aBMD was 71%. While there was no significant difference between patients with upper limb fractures and those with PFFs in the %YAM of spine aBMD (both at 85%), there was a significant difference in the %YAM of the femoral neck aBMD, at 79% and 65%, respectively. For bone turnover biomarkers, the mean serum total P1NP concentration was 125 ng/mL, the mean serum TRACP-5b concentration was 305 mU/dL, and the mean serum 25(OH)D concentration was 13.5 ng/mL. The serum total P1NP levels were significantly lower at 69.0 ng/mL and 25(OH)D levels were significantly greater at 15.8 ng/mL in the upper limb group than in the PFF group (at 169 ng/mL and 11.7 ng/mL, respectively), whereas serum TRACP-5b levels were not significantly different between the two groups (261 mU/dL and 338 mU/dL, respectively) (p<0.05).

**Table 2:** Clinical outcomes evaluated in this study The data are presented as the means (minimum-maximum) or percentages. *Between upper limb fracture and lower limb fracture. **The number of new therapeutic interventions for osteoporosis in our hospital per the first diagnosis of osteoporosis. PHF, proximal humerus fracture; DRF, distal radius fracture; PFF, proximal femoral fracture; BMD, bone mineral density; YAM, young adult mean; aBMD, areal bone mineral density; total P1NP, total procollagen type I propeptide; TRACP-5b, tartrate-resistant acid phosphatase 5b; 25(OH)D, 25-hydroxy-vitamin D

	Total	Upper limb fracture			PFF	p Value*	t-Value
		Total	PHF	DRF			
BMD test conducted, number (%)	67 (58.2)	28 (54.9)	2 (20.0)	26 (63.4)	39 (60.9)	0.570	-
Blood test with bone turnover biomarkers conducted, number (%)	47 (40.9)	20 (39.2)	3 (30.0)	17 (41.5)	27 (42.2)	0.707	-
The first diagnosis for osteoporosis in our hospital, number (%)	82 (71.3)	18 (35.1)	2 (20.0)	16 (39)	64 (100)	0.430	-
New therapeutic intervention for osteoporosis in our hospital, number (%)**	39 (47.0)	15 (83.3)	2 (100)	13 (81.3)	24 (37.5)	<0.001	-
%YAM of spine aBMD (mean), %	86 (48-126)	85 (56-118)	76 (73-78)	88 (56-118)	85 (48-126)	0.733	-0.343
%YAM of femoral neck aBMD (mean), %	71 (41-104)	79 (56-104)	78 (69-86)	79 (56-104)	65 (41-85)	<0.001	-4.66
Serum total P1NP (mean), ng/mL	125 (19.6-788)	69.0 (20.4-240)	126 (39.3-240)	59.6 (20.4-133)	169 (19.6-788)	0.0224	2.36
Serum TRACP-5b (mean), mU/dL	305 (117-1060)	261 (122-633)	257 (122-345)	262 (127-633)	338 (117-1060)	0.116	1.60
Serum 25(OH)D (mean), ng/mL	13.5 (4.3-27.9)	15.8 (8.4-21.5)	15.4 (11.2-18)	15.9 (8.4-21.5)	11.7 (4.3-27.9)	0.0175	-2.50

The details of medications for osteoporosis are shown in Table 3. Although activated vitamin D3 (n=33, 28.7%) and bisphosphonates (n=22, 19.1%) were more frequently used among all patients, there was no significant difference in medications between patients with upper limb fractures and PFFs.

**Table 3:** Details of medications used for osteoporosis The data are presented as numbers (percentages). Note: Some patients used two or more medications, and the numbers include medications that were administered by other clinics or hospitals before fractures. *Between upper limb fracture and PFF. PHF, proximal humerus fracture; DRF, distal radius fracture; PFF, proximal femoral fracture; SERM, selective estrogen receptor modulator

Medications	Total (n=115)	Upper limb fracture			PFF (n=64)	p- Value*
		Total (n=51)	PHF (n=10)	DRF (n=41)		
Bisphosphonates	22 (19.1)	9 (17.6)	2 (20.0)	7 (17.0)	13 (20.3)	0.999
Denosumab	7 (6.1)	2 (3.9)	0 (0)	2 (4.9)	5 (7.8)	0.46
Activated vitamin D3	33 (28.7)	10 (19.6)	4 (40.0)	6 (14.6)	23 (35.9)	0.0958
SERM	4 (3.5)	0 (0)	0 (0)	0 (0)	4 (6.3)	0.128
Daily teriparatide	1 (0.9)	1 (2.0)	0 (0)	1 (2.4)	0 (0)	0.443
Once or twice-weekly teriparatide	3 (2.6)	1 (2.0)	0 (0)	1 (2.4)	2 (3.1)	0.999
Romosozumab	3 (2.6)	1 (2.0)	0 (0)	1 (2.4)	2 (3.1)	0.999

There were only two (1.7%) patients with secondary fractures during the study period. One was affected by PHF four months after primary DRF, who was administered osteoporosis treatment before the primary fracture by another clinic. Another was affected by secondary contralateral PFF two years after the first PFF, who had not been administered osteoporosis treatment because of the patient’s rejection.

## Discussion

The Inba region of Chiba Prefecture in Japan, where our hospital is located, has approximately 700,000 residents, but there are not many medical facilities for primary or secondary care compared with the nationwide average in Japan [14], especially in the eastern part of the region, which is the closest area to our hospital [15]. It was considered relatively difficult for citizens to access medical care before our hospital opened. According to the ROAD study, which involved a large cohort of Japanese urban and rural areas, the number of patients with osteoporosis in Japan was estimated to be 12.8 million [10], which is approximately one-tenth of the total Japanese population. The area of our hospital has a developing aging society and is considered to be a typical rural area in Japan, and the number of potential patients requiring therapeutic intervention for osteoporosis in this area is estimated to be seventy thousand. Although diagnostic and therapeutic interventions for osteoporosis should be administered to as many potential patients as possible, our study revealed the low rate of osteoporosis intervention for patients with fragility fractures before they came to our hospital in this area.

This study revealed that 100% of the patients with PHFs and 81.3 % of the patients with DRFs, who were newly diagnosed with osteoporosis in our hospital, were newly administered the intervention for osteoporosis in our hospital, which is greater than that of the previous reports [2]; however, the intervention rate in patients with PFFs was 37.5%, which is also higher than that the previous result [9], but it is not much. The intervention rates in patients with PFFs were lower than those with upper limb fractures in our hospital because most patients with upper limb fractures were admitted to our outpatient clinic for at least several months after surgery, and it was relatively easy to do an intervention for osteoporosis by our hospital. In contrast, most patients with PFFs should be transferred to another hospital for postoperative rehabilitation in the early postoperative period due to the system of Japanese national medical insurance, which is a unique national medical insurance system, and the cost of each medical practice that can be charged to patients is determined by a ministerial ordinance, making it difficult for us to provide them with osteoporosis therapeutic intervention and to follow them up in our hospital.

The rate of osteoporosis intervention before admission to our hospital in patients with upper limb fractures was lower than those with PFFs. It was presumed that it was because the mean age of the DRF group was younger than that of the PFF group. Additionally, since some patients with PHFs and DRFs had more than 80% of YAM in BMD and were not included in the Japanese criteria with the guideline for osteoporosis [13], the number of patients with PHF and DRF who were diagnosed with osteoporosis after surgery was lower than that of patients with PFF. This might cause a low number of patients who were newly diagnosed with osteoporosis after upper extremity fractures, resulting in a higher rate of therapeutic intervention than that of the PFF group.

BMD was reported to be highly associated with fracture risk of the same site [16], and proximal femoral aBMD, not spine aBMD, was reported to be a predictor of all fractures [17]. In our study, although there was no significant difference in spine aBMD between the upper limb fracture group and the PFF group, there was a significant difference in femoral neck aBMD. Our study revealed the relevant results as the previous reports and found that proximal femoral aBMD is a stronger predictor than spine aBMD for risk assessment of non-vertebral fractures, including PFF.

High levels of serum P1NP, in addition to high levels of serum type 1 collagen C-terminal telopeptide (CTX-1) and type 1 collagen N-terminal telopeptide (NTX-1), which are one of the representative bone resorption markers, were significantly associated with PFF in an Asian cohort study [18]. Moreover, high levels of serum bone resorption markers were reported to be a risk of any fracture [19]. Furthermore, in a previous study investigating the relationship between DRF and the serum bone turnover biomarkers and 25(OH)D, high levels of serum CTX-1 were associated with an increasing risk of DRF, whereas high levels of serum P1NP and bone alkaline phosphatase (BAP), which is one of the representative bone formation markers, were not associated with DRF [20]. Thus, our result that P1NP, not TRACP-5b, was significantly associated with the PFF group, not the upper limb fracture group, and there was no difference in TRACP-5b between the two groups, is consistent with the results of these previous reports.

It was reported that more than half of Japanese women had vitamin D deficiency and it affected the risk of fragility fractures over the subsequent five years [21]. In our study, the mean serum 25(OH)D levels of all participants were very low, at 13.5 ng/mL, as vitamin D deficiency, which is consistent with the previous result. Levels of serum 25(OH)D were reported not to be associated with DRF [20], and serum 25(OH)D levels were significantly lower in the PFF group than in the upper limb fracture group in this study. This previous result and ours indicate that low serum 25(OH)D levels may be associated with a higher risk of PFFs than upper limb fractures, including DRFs.

In 2006, the special cost of the Clinical Pathway with Regional Alliance (CPRA) system of the PFF among hospitals and clinics was introduced in Japan. This has promoted the creation of regional clinical pathways and cooperation between hospitals and clinics in various regions all over the country, and a positive CPRA result was reported [22]. According to this report, creating a clinical pathway and cooperating with regional medical institutes can improve the rate of osteoporosis diagnosis and therapeutic intervention, and we will develop our CPRA between our hospital and nearby medical institutes. Moreover, we are establishing a fracture liaison service (FLS) team to increase the rate of osteoporosis diagnosis and treatment intervention. There are many reports about the effectiveness of FLSs, and a previous report from a rural regional hospital in Japan, similar to our hospital, showed that the introduction of FLS dramatically increased the intervention rate of osteoporosis treatment [23]. Currently, we are preparing to introduce FLS in our hospital to related professionals.

This study has several limitations. First, since this is a retrospective study, there might be variations in the diagnosis and treatment process of the physicians who attended each patient, which might have affected the results. Second, the statistical power might be insufficient because of the relatively small number of participants in this study. Third, although bone turnover markers generally increased after fracture occurrences [24], we measured them after fractures, and the measurement time differed for each patient. Thus, our values of bone turnover markers might not be reflected in the usual values in each patient. Last, the whole of the study period was under the COVID-19 pandemic, and the medical system and patients' daily activities and frequency of visits to the clinics and hospitals might have differed from the ordinary time.

## Conclusions

In conclusion, the intervention rates for the diagnosis and treatment of osteoporosis after surgery for non-vertebral fractures in our hospital, especially in upper limb fractures, were greater than that in previous reports in Japan, and the upper limb fracture group had higher femoral neck aBMD, lower serum P1NP, and higher serum 25(OH)D than the PFF group. Since the intervention rate for osteoporosis in the PFF group was higher than those of the previous report, but never too much, there is still room for improvement in the medical systems of our hospital. Further increasing the intervention rate through regional collaborative clinical pathways and FLS team establishment is necessary.
